# Hydrogel based tissue engineering and its future applications in personalized disease modeling and regenerative therapy

**DOI:** 10.1186/s43088-021-00172-1

**Published:** 2022-01-04

**Authors:** Shikha Chaudhary, Eliza Chakraborty

**Affiliations:** 1 SRM Institute of Science & Technology, Chennai, Tamil Nadu 603203 India; 2 Medical Translational Biotechnology Lab, Prof of Department of Biotechnology, Head of the Department of DST-Fist Center (Sponsored By Ministry of Science & Technology, Government of India), Meerut Institute of Engineering and Technology (MIET), Meerut, Uttar Pradesh 250002 India

**Keywords:** 3D cell culture, Hydrogel, Extra Cellular Matrix, Organoid, Stem cells, Tissue Engineering, Vascularization, Personalized medicine, Disease models

## Abstract

**Background:**

Evolution in the in vitro cell culture from conventional 2D to 3D technique has been a significant accomplishment. The 3D culture models have provided a close and better insight into the physiological study of the human body. The increasing demand for organs like liver, kidney, and pancreas for transplantation, rapid anti-cancer drug screening, and the limitations associated with the use of animal models have attracted the interest of researchers to explore 3D organ culture.

**Main body:**

Natural, synthetic, and hybrid material-based hydrogels are being used as scaffolds in 3D culture and provide 'close-to-in vivo’ structures. Organoids: the stem cell-derived small size 3D culture systems are now favored due to their ability to mimic the in-vivo conditions of organ or tissue and this characteristic has made it eligible for a variety of clinical applications, drug discovery and regenerative medicine are a few of the many areas of application. The use of animal models for clinical applications has been a long-time ethical and biological challenge to get accurate outcomes. 3D bioprinting has resolved the issue of vascularization in organoid culture to a great extent by its layer-by-layer construction approach. The 3D bioprinted organoids have a popular application in personalized disease modeling and rapid drug development and therapeutics.

**Short conclusions:**

This review paper, focuses on discussing the novel organoid culture approach, its advantages and limitations, and potential applications in a variety of life science areas namely cancer research, cell therapy, tissue engineering, and personalized medicine and drug discovery.

**Graphical Abstract:**

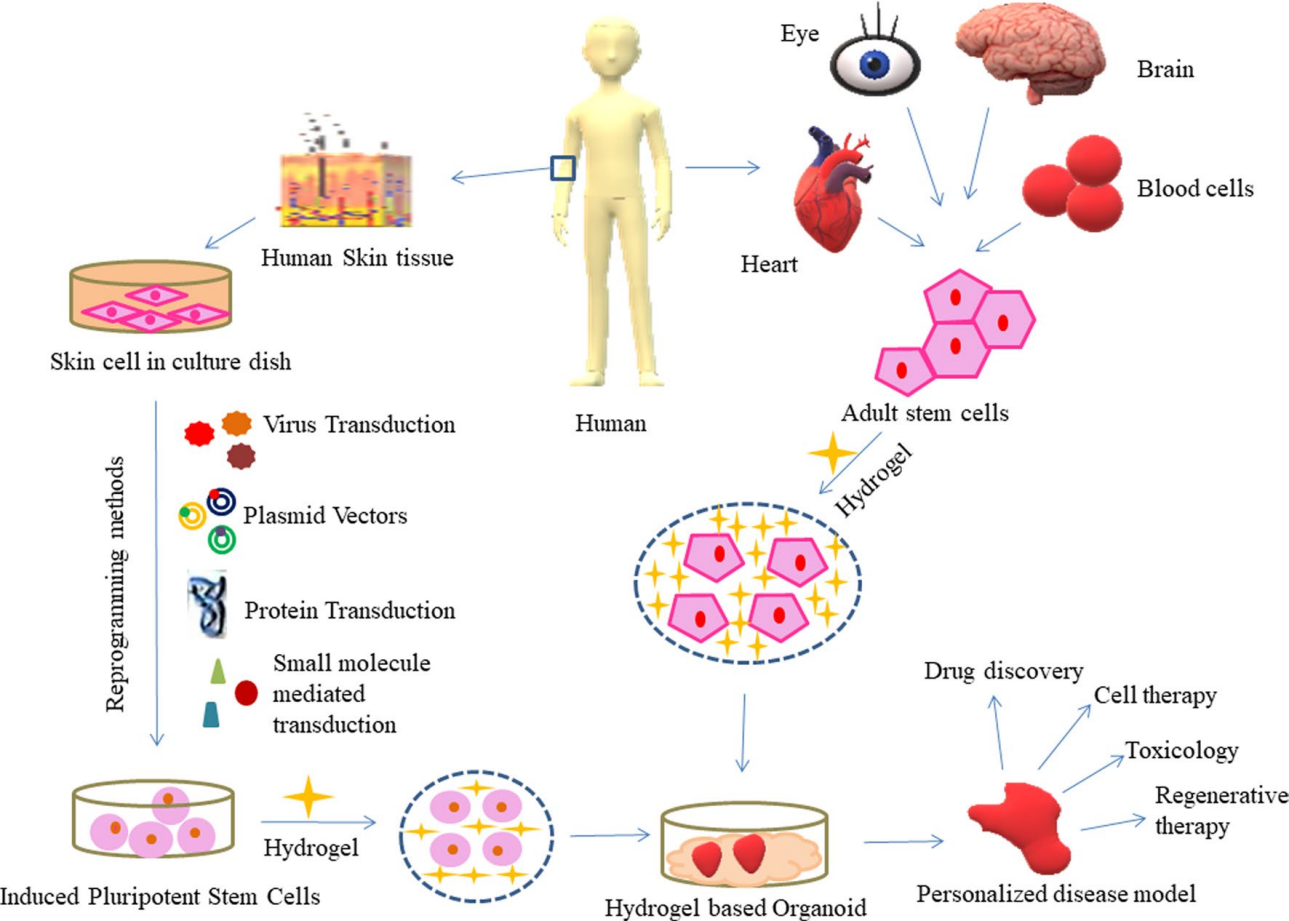

## Background

A cell is a fundamental unit of an organism’s structure consisting of genetic material & organelles for their destined functions. Cells communicate via cell–cell interaction to form a functional organ. Mammalian cell culture is a principal technique used for the analysis of drug activity, metabolism, and toxicity in vivo [1]. The first in vitro mammalian cell culture technique was performed by an American embryologist Ross Granville Harrison in the first decade of the twentieth century and the technique was named Harrison's hanging drop technique [2]. Different types of cells require different types of environments to grow in vitro. Primary cells and stem cells grow anchorage-dependent and blood cells and secondary cell lines grow in suspension culture or anchorage-independent. Cell culture can be performed in vitro by any of the two established methods: two-dimensional cell culture (2D) or three-dimensional cell culture (3D). Both the methods have their respective advantages, disadvantages, and limitations. 2D cell culturing is a conventional method used to date for establishing a cell line and various research experiments designed in vitro, however, a new approach of 3D cell culture is advantageous as the in vivo conditions can be mimicked by using various types of cellular matrix and produce an artificial organ [3]. The 3D cell culture models prepared in vitro are a potential alternative to animal experiments [4]. As the 2D cell culture provides a monolayer of cells as the result, 3D cell culture can be performed either using a scaffold or by using a scaffold-free technique [3]. 3D cell culture has helped to make the cancer researchers understand the cancer cell microenvironment, the role of chemoresistance in cancer cells survival, and unlimited growth in a small time, and also it provides an opportunity to develop new highly effective anticancer drugs with minimum risk of side effects or other complications [54]. The increasing chronic shortage of organs like liver, kidney, and pancreas for transplantation is leading to more deaths in comparison to other diseases like cancer (colon cancer, blood cancer, prostate cancer, and breast cancer), and neurodegenerative diseases.

A scaffold is a material that possesses the ability to mimic the extracellular matrix present in vivo to support the cellular structure and provide them with a suitable microenvironment. Two types of scaffolds are available natural and synthetic. 3D cell culture is proving as a novel approach in cancer research, stem cell, drug metabolism, tissue regeneration, etc.

Tissue engineering is a combination of life science, biochemistry, and engineering to understand the structure and function of mammalian tissue and develop synthetic substitutes that can mimic the original and can be used in the regeneration or repair of damaged or lost tissue in accidents or disease [5]. Tissue engineering is also a promising solution to organ donation and its related complications [5]. Scaffolds that are biocompatible, biodegradable, and have good mechanical strength are preferred for tissue engineering. Typically there are 2 classes to classify the methods for scaffold manufacturing and fabrication: Conventional fabrication techniques and Rapid prototyping [5]. Among the various methods, Solvent Casting and Particle Leaching (SCPL), Freeze-drying, Electrospinning, and 3D bioprinting techniques are commonly used for scaffold production [3]. Bioprinting is also being used for organ printing, in the fabrication of in vitro tissue models for purposes like drug screening, disease modeling, and several other applications [53]. In the date, continuing process of 3D cell culture-based research and development, several biomaterials such as patterned glass substrates, elastomeric films, hydroxyapatite ceramics, hydrogels, and fibrillar foams have been used as an alternative of physical scaffold for cells [6].

Hydrogel has been used for decades in 3D cell culture-based research applications. Their ability to contain high water content and high flexibility make them alike to the natural tissues [7]. Hydrogels are swollen due to the absorbed water content. Water is absorbed by the functional groups attached with the polymeric backbone and does not dissolve since intermolecular or interfibrillar cross-link structures are formed between the network chains formed by polymer molecules or fibrillar proteins respectively [7, 8]. A novel plant-based nanocellulose hydrogel has been developed at a low cost and the mechanical properties observed were similar to the popular standard Engel-Holm Swarm matrix derived from the basement membrane of mouse sarcoma [59]. Nanocellulose and alginate are the leading potential biomaterials for 3D bioprinting [62].

The researchers have been continuously developing new protocols in the field of tissue engineering technology and diseases like cancer, autoimmune deficiencies, diabetes, organ failure, and skin injuries are the few names that highly demand personalized models for the cure. In the development of engineered tissues, lack of vascularization has been a constant challenge for the researchers due to which the application of invitro-developed models is very limited. Here, we aim to explore hydrogel-based tissue engineering methods to develop a personalized disease model which may help in discovering the new avenues in the field of regenerative therapy. The paper discusses all the challenges and their most suitable solutions in developing hydrogel-based engineered tissues further indicating the possible applications of the developed tissues

## Main text

### Hydrogel categorization

**Fig. 1:**
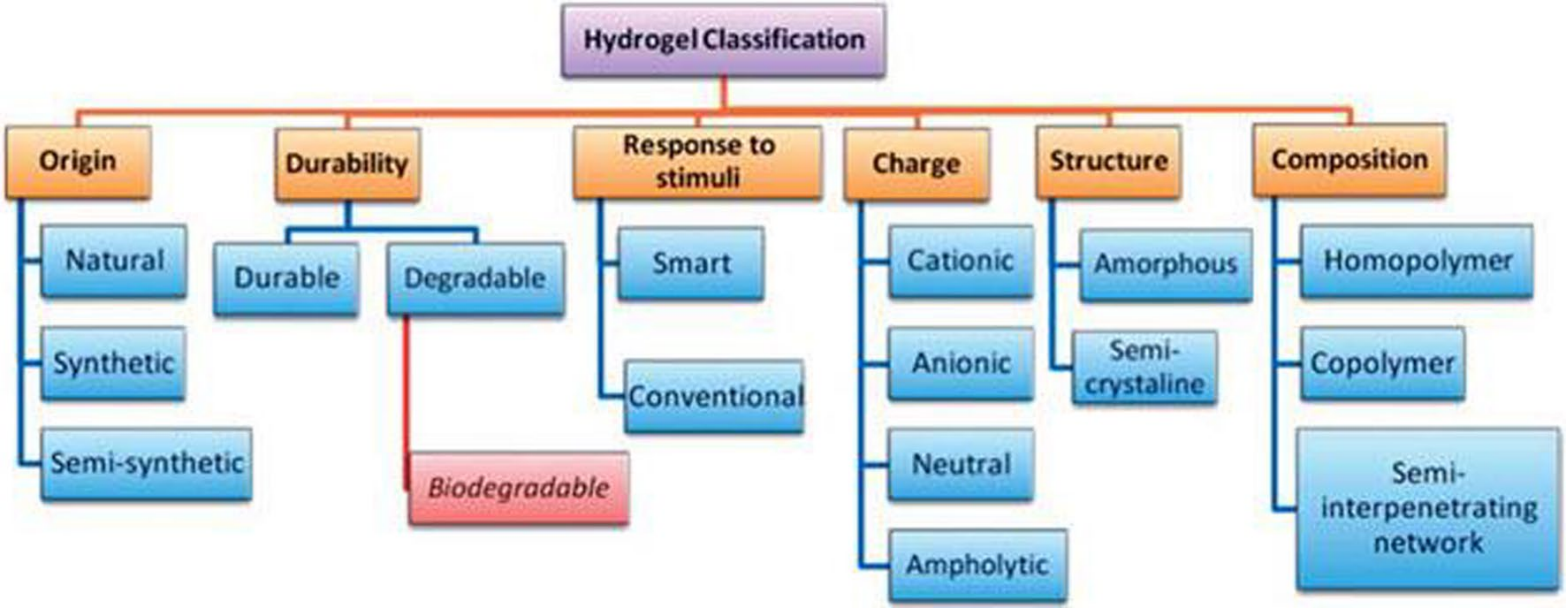
Classification of hydrogel. Courtesy of Malpure et al. [65]

Hydrogels have helped in revealing the elemental phenomenon regulating cell behavior and providing tools for the expansion and directed differentiation of various cell types in ways that are unique to 3D cell culture substrates [9]. The major classification of hydrogels can be given as natural, synthetic, and hybrid material-based hydrogels. Table 1 refers to the detailed categorization of natural and synthetic hydrogels and Fig. 1 presents hydrogel classification based on various other parameters.

**Table 1:** Classification of natural and synthetic hydrogels: material, source, characteristic, application and reference

Material	Source	Characteristic	Application	References
*1. Natural (Animal Based)*				
Alginate	Brown Seaweed	Low cytotoxicity	Cell-laden bio inks in 3D-bioprinting	[10]
		Less Immunogenicity		
		Ability to cross link		
		Biodegradable		
Gelatin	Fish, Cattle, Bone, Skin, Pig, etc	Biocompatible	Bone tissue engineering	[10]
		Biodegradable		
		Low antigenicity		
		Non-toxic		
		Low mechanical stability		
Collagen	Rat tail, Bovine tendon, Jelly fish, Porcine, etc	Low viscosity	Tissue engineering and drug delivery	[5, 10]
		Low immunogenicity		
		Biocompatible		
Hyaluronic Acid	Human and Bacterial fermentation	Immunoneutral. Polysaccharide	Dynamic cell patterning	[9, 11]
		Non-adhesive		
Silk Fibroin	Silkworm (Bombyx mori)	High strength and stiffness	Useful in Bone tissue engineering	[10]
		Biocompatible and biodegradable		
Fibrin	Human Plasma	Biocompatible	Fibrin-hybrid constructs useful cartilage, cardiac, smooth muscle cultures and drug delivery systems	[5, 9, 13, 15]
		High cell affinity		
		Rapid degradability		
		Weak mechanical strength		
*2. Natural (Plant Based)*				
i. Plant—lignocellulose materials based				
Cellulose	Plant cell and natural fibres	Highly crystalline	Application in biomedical	[20]
		Low water solubility		
		Slow biodegradability in vivo		
Hemicellulose	Plant cell wall	Biocompatible	Application in biomedical	[20]
		Non-toxic		
		Biodegradable		
Lignin	Plant cell wall	Biodegradable	Drug delivery and tissue engineering	[20]
		Hydrophobic		
		Antioxidant		
		Antimicrobial		
		High global availability		
ii. Plant—polysaccharide Based				
Starch	Chloroplast and amyloplast of the plant	Hydrophilic	Tissue engineering, Drug delivery, Agricultural usage, water treatment, Food industry	[20]
		Biocompatible		
Pectin	Fruit peels and pulp, sugar beet, sunflower heads	High gelling capacity	Drug delivery and tissue engineering	[20]
		Hydrophilic		
iii. Plant—gum based				
Natural gums	Plant exudation, seed endosperm, tree exudation	Excellent biocompatibility and biodegradability	Tissue engineering, Drug delivery, Can be used for oral delivery platform	[20]
		Mimic ECM		
		Renewable		
		Edible		
iv. Plant protein based				
Soy	Soybean	Biocompatible	Tissue engineering, drug delivery, regenerative medicine	[20]
		Bio reactive		
		Biodegradable		
		High water holding capacity		
Zein	Corn kernels	Hydrophobic	Tissue engineering, drug delivery, regenerative medicine	[20]
*3. Synthetic*				
Poly Ethylene Glycol	By-products of petroleum	Compositionally consistent	In combination with natural polymer suitable as scaffold	[12]
		Non-toxic		
		Hydrophilic		
Poly Vinyl Alcohol	Hydrolysis of Poly vinyl acetate	Lack biologically active sites	In combination with natural polymer suitable ﻿as scaffold	[12]
Poly Acrylic Acid		Low Mechanical Strength	In combination with natural polymer suitable ﻿as scaffold	[12]

### Properties of hydrogels

1. Swelling properties: In standard hydrogel systems, drug release depends on swelling or contraction of the hydrogel and diffusion of the drug through the polymer network. Temperature, pH, ion concentration, the molecular weight of polymers, and crosslinking capability are important parameters that participate in the collapse or phase transfer of the hydrogel [66].
2. Mechanical properties: These properties of scaffold play an important role in regulating cell behavior. Stiffness, elasticity, tensile strength, stress relaxation, self-healing, and degradation, can be controlled at various levels to meet specific requirements for specific applications [67].
3. Biological Properties: Biocompatibility, non-toxicity, non-carcinogenicity, non-inflammatory, and biodegradability are the main requirements that all types of hydrogel should possess [66].

### Organoids to supplant animal models in research

3D cell culture technique is a rapidly developing area of biotechnology intending to replace the animal models been used for decades for drug discovery, cancer research, and other disease-related researches. So far the scientists have succeeded to prepare skin, cornea, blood vessels, thyroid organoids, liver organoids, stomach organoids, and many more using the 3D cell culture [13, 17]. To supersede animal models use in research the 3D cell culturing can utilize the ‘replace’ and ‘reduce’ strategies from the 3R principles for animal use proposed by Russel and Burch in 1959 [13].

**Fig. 2:**
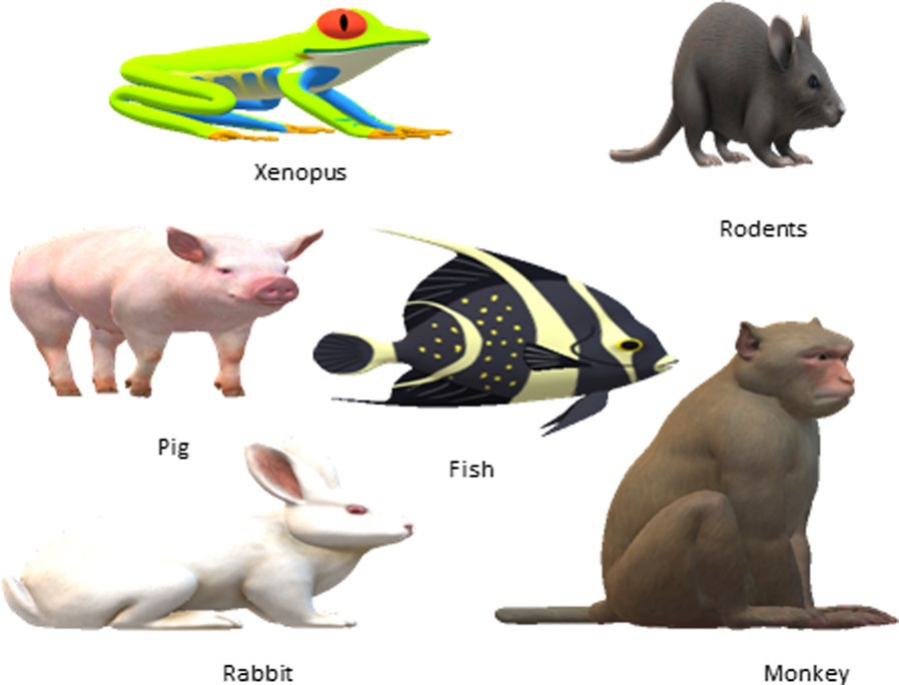
Various animal models used for research and therapeutic studies: Xenopus, rodent, rabbit, and fish mostly preferred because of their small size, easy maintenance in laboratories, small gestation period, and a high number of live young. Genetic similarity between these organisms and humans makes them a preferable choice as human disease models. Pig and Monkey are used in research due to their organ size, and structure. The development of new genome modification tools resulted in regaining the interest of researchers towards large size non-rodent and non-human species

Organoids are the self-organized 3D structures derived from embryonic or adult stem cells and mimic the structure and functionality of the tissue of origin or the tissue from which they are derived [14]. Organoids are the outcomes of anchorage-dependent technologies (Scaffold based 3D cell culture models) and spheroids are the result of anchorage-independent technologies (Scaffold free 3D cell culture models). Regulation of key signaling pathways, standardization of media formulation, and expansion in three dimensions by aggregation or embedding the cells in the scaffold are the three major steps followed to develop an organoid [34]. Cells implanted into the scaffold matrix and utilizing the chemical and physical properties of the scaffold cells start aggregation, proliferation, and migration [16]. Organoids are small in size (typically ≤ 3 mm in diameter) and are stable model systems of organs and tissues that are amenable to long-term cultivation and manipulation [17]. A brief comparative analysis of Organoids and Animal models is described in Table 2 and various animal models presented in Fig. 2.

**Table 2:** Comparative study of 3D cell culture over animal models

Parameter	3D cell culture (organoid)	Animal model	References
Cost	Low	Expensive	[13, 16, 17]
Immunogenic response	Incorporation of immunogenic components under research	The response occurs in normal animal models however immunodeficient models lack such responses	[13, 17]
Vascularization	Not present	Reflect to in vivo	[13, 17]
Ethical concerns	No ethical issues because no animal testing is required. Only the use of animal serum raises concerns for animal welfare and human biosafety	Ethical concerns required to be addressed	[13]
Experimental complexity	Less complex	Higher organisms are used therefore high complexity	[13, 17]
Human in vivo imitation	Imitate the source tissue or organ	Does not imitate due to variation at the genetic level	[13, 17]
Genetic expression	Reflective to humans	Differ from humans	[13]
Cell microenvironment	Lack microenvironment, therefore, scaffolds used	Present naturally	[16]
Reproducibility	Low since scaffolds are used	Not satisfactory	[13, 17]

### Techniques for hydrogel preparation

Scaffolds and matrices are one of the important components in the preparation of 'Closer-to-in vivo' 3D cell culture systems [18]. These scaffolds are generated using various natural, synthetic, or composite polymers and each, natural or synthetic polymer has its characteristics (Refer Table 1). Hydrogel has been identified as distinctly effective matrices for 3D cell culture and restates the aspects of the cellular microenvironment [19]. The natural gels namely collagen, fibrin, alginate, gelatin are biocompatible and biodegradable since they are obtained from natural sources but many times they are not acceptable for the cell culture process due to low viscosity, low mechanical strength, and various other reasons specific to research therefore composite polymers or Natural-synthetic hybrid polymers are most preferred for research and clinical application purposes. In vitro, scaffolds serve as the backbone to prepare a complex miniature (organoid) by providing the required mechanical support and also support cell–cell interaction and cell matrices interaction which leads to proper cell adhesion, proliferation, differentiation, morphology, and genetics. Hydrogel preparation completes in 2 major steps: Manufacturing (Polymerization) and Fabrication as described below:

- **Polymerization**

i. **Physical cross-linking**

Non-covalent interactions like ionic interactions, hydrogen bonding, hydrophobic interactions, crystallite formation, induce physical cross-linking between the polymers. This method is advantageous over chemical cross-linking because of less toxicity and often reversible processes however, the inflexibility towards gelation time, pore size, and chemical functionalization mark the limitations of physical cross-linking techniques [6]. Ionic interactions occur between oppositely charged groups by electrostatic force. The alginate, cellulose, chitosan-based hydrogels are commonly prepared by the ionic interaction method [21]. Usually single hydrogen bond forms a weak interaction but multiple hydrogen bonds can contribute to the formation of hydrogels. The formation and destruction of hydrogen bonds are sensitive to various parameters like pH, temperature, or type of solvent [21]. Natural polymers such as gelatin, collagen, agar, and starch can easily form hydrogels by hydrogen bonding in presence of side groups such as –OH, -COOH, and –NH_2_ [21]. Hydrophobic interaction induces high uptake of water and swelling up, for the formation of hydrogel structures. Chitosan and dextran hydrogels are prepared using the hydrophobic interaction method [22].

ii. **Chemical cross-linking**

Chemical cross-linking is an irreversible process. Both natural and synthetic hydrogels can be prepared by covalent bonding which is induced by processes like chain-growth polymerization, condensation polymerization, or by sequential addition of cross-linking agents [22]. This method can help to perform polymerization following a controlled and precisely managed procedure, potentially in a spatially and dynamically defined manner [26].

iii. **Irradiation based cross-linking**

Ionizing radiations such as X-rays, gamma rays, accelerated electron, ion beam, high energy ultraviolet rays are used for the polymerization reaction. The cross-linking process is controlled and intentional modifications are implied by controlling the parameters like exposure time of radiation, frequency, temperature, and pressure [22].

iv. **Enzyme based cross-linking**

Enzymes are proteins of high molecular weight and work as specific, versatile, and very effective biological catalysts. The enzymatic cross-linking method is a potential alternative to the chemical cross-linking technique as the polymerized hydrogel would be non-toxic and biocompatible. However, the substrate specificity and high cost are the limitations of this technique. Transglutaminases, tyrosinase, lysyl oxidase, phosphatases, and horseradish peroxidase (HRP) and hydrogen peroxide (H_2_O_2_) are generally being used for the synthesis of various types of hydrogels [23]. Hydrogels prepared using enzyme cross-linking have great application in the field of tissue engineering, drug delivery, cartilage treatment. For example, to cure a skin defect, hyaluronic acid, carboxylated chitosan, and human-like collagen-based hydrogel was synthesized using transglutaminase as a cross-linking agent and the hydrogel formed was successful to mimic the extracellular matrix of the human body [24]. Another successful application of the enzymatic crosslinking method was observed when bone-marrow-derived mesenchymal stem cells (BMSCs)—laden hydrogel was synthesized with hyaluronic acid-tyramine and chondroitin sulfate-tyramine where the reaction was catalyzed by hydrogen peroxide and horseradish peroxidase and the resulting injectable BMSCs-laden hydrogel could serve as an efficient 3D scaffold for bone repair and regeneration [25].

b. **Fabrication**

i. **Freeze drying**

Freeze drying also known as lyophilization is one of the most commonly preferred fabrication techniques for the creation of hydrogel scaffolds for 3D cell culture. A polymer solution is mixed in an organic solvent and water mixture creating an emulsion. The emulsion is cooled at − 70 °C, resulting in a solid solvent that undergoes a sublimation process where the solvent evaporates and a porous solid scaffold is obtained as the final product. This technique is advantageous because the pore size is manageable to be controlled by varying the freezing temperature (− 10 °C to − 70 °C) [5].

ii. **Solvent casting and particle leaching**

This method is also known as Porogen leaching [22]. Based on the desired pore size, salt particles are selected and mixed with solvent and this mixture is used to dissolve the polymer solution. Once the desired shape is achieved, the mold is freeze-dried leading to sublimation of solvent, and a matrix containing salt particles remains. The matrix containing salt particles undergo leaching and finally, a high porosity structure is obtained. 50–90% porous scaffolds are developed by this method and this technique is relatively easy and low cost [5].

iii. **Electrospinning**

One of the reliable, economical but complicated methods is electrospinning. A standard electrospinning system consists of 4 main components: a spinner with a syringe pump, a metallic needle, a high voltage power supply, and a grounded collector [5]. A polymer solution remains filled in the syringe pump, when a current is applied to the pump it leads to electric repulsion within the polymer solution, which jets the polymer out of the nozzle tip as thin filamentous strands, and these fibers are collected by the rotating target collector based on the requirement of scaffold properties [22].

iv. **Bioprinting**

In a short period, bioprinting technology has gained high importance in the field of tissue engineering and regenerative medicine for generating 3D structures. This technique has an advantage over freeze-drying, porogen leaching, and electrospinning methods because no organic solvents, residues of which would be toxic to cells are used [27]. In 3D bioprinting, layer-by-layer precise positioning of biological materials, biochemical and living cells, with spatial control of the placement of functional components, is used to fabricate 3D structures [28]. Biomimicry, autonomous self-assembly, and mini tissue building blocks are the three approaches to 3D bioprinting. Tissue bioprinting technologies have been classified into 3 main classes: inkjet, microextrusion, and laser-assisted printing. For each of these, the ‘bioinks’ are selected based on the ink’s rheology, biocompatibility, viscosity, and crosslinking chemistry [53]. ‘Bioinks’ are the biomaterial required for the loading of cells and hydrogels are the most preferred because of their high water content. Polycaprolactone (PCL), polydimethylsiloxane (PDMS), and PEG are also been widely used as biocompatible inks where PDMS with soft lithography evolved to develop an organ-on-a-chip with microchannels to manipulate the extremely small amount of fluid flow [57]. To fulfill the need for bioinks that can directly print cell-laden constructs, the agarose–alginate mixtures were analyzed in comparison to pluronic, and the agarose-alginate combination showed the greatest potential as an effective bioink for additive manufacturing of biological materials for cartilage tissue engineering [60]. Plant-based biomaterials like proteins, extracts, secondary metabolites, and polysaccharides are cost-effective, biocompatible, and natural alternatives to animal or synthetic biomaterials used for fabrication using bioprinting for tissue engineering applications [61].

Inkjet bioprinting is a noncontact technique that uses picolitre bioink droplets to construct 2D or 3D structures layered onto a substrate [5]. Droplet size (< 1 pl to > 300 pl in volume) and deposition rate (1–10,000 droplets per second) can be controlled electronically [28]. Inkjet bioprinting is a low-cost, high speed, high resolution, and biocompatible technique. Low viscous materials such as thrombin, CaCl_2_, saline, and fibrogen have been used as bio-inks for inkjet printing since high viscosity materials cannot generate picolitre droplets [27].

Microextrusion bioprinters usually consist of a temperature-controlled material-handling and dispensing system and stage, with one or both capable of movement along the x, y, and z axes. The fiber-optic-based light source could be used to eliminate the deposition area for photo-initiator activation and photographer's activity and as a piezoelectric humidifier and a video camera to command and control for *x*–*y*–*z*. Some systems use more than one print head to make the serial dispensing of several materials easy without retooling [5, 28]. In this process, continuous beads of material are produced rather than liquid droplets. Extrusion printing is generally used bioprinting method in tissue engineering because of the following certain advantages: the ability to print one construct with multiple printheads and materials; print constructs with certain differences in biomaterials, cell types, cell densities, and signaling molecules; ability to print with higher cell densities [55, 56]. Lack of available bioinks is the most commonly cited limitation of extrusion bioprinting [56].

The laser-assisted bioprinting (LAB) technique works on the principles of laser-induced forward transfer. The LAB device consists of the following components: pulsed laser beam along with a focusing system; a ‘ribbon’ with donor transport support covered with a laser-energy-absorbing layer of gold or titanium and a layer of biological material prepared in a liquid solution; and a receiving substrate facing the ribbon. LAB directs focused laser pulses over the absorbing layer of gold or titanium of the ribbon that leads to the generation of a high-pressure bubble which further sets in motion the biological material towards the collector substrate. LAB does not counter clogging because it is a nozzle-free technique and also it can print mammalian cells without affecting the cell function and viability.

### Challenges and their alternatives in organ development on petridish

For in vitro organ development, the human organs can be divided into 4 categories based on their structure complexity: zero complexity flat structures (example—skin), tubular structure (example—blood vessels, trachea, and esophagus), hollow organs (example—stomach, intestines, gall bladder, urinary bladder, and bile ducts), and solid organs (example—liver, heart, kidney, lungs, pancreas [35]. A young woman with an acute bronchial disease in 2008 received the first successful stem cell-based tracheal graft where decellularized donor tracheal graft was repopulated with autologous mesenchymal stem cell-derived chondrocyte cells and survived without any immunosuppressants [36]. Development of organoids on hydrogel scaffold has been a challenge mainly due to lack of vascularization which brings in other limitations like small size, slow proliferation and differentiation rate, lack of immune system, limited growth, and development of the necrotic core. Therefore setting up the vascularization structures in organoids is necessary to allow not only oxygen, nutrient, and waste exchange but also provide the proper immune system. The characteristic of limited growth is hypothesized to be due to 2 reasons: a transformation from undifferentiated stem cell state to differentiated cell state and the loss of cell viability in the organoid core leading to necrosis upon reaching a certain size [37]. Various approaches like additive manufacturing (example—bioprinting, digital micro-mirror patterning, and direct 2-photon fabrication), sacrificial networks, subtractive fabrication, and spontaneous vascularization (implantation of an organoid into highly vascularized tissue or co-culturing organoid near endothelial cell monolayer for angiogenesis) are being adopted to develop vascularized organoid [37]. Pericytes, the perivascular cells that surround the endothelial cells in capillaries, venules, and arterioles were recently isolated from induced pluripotent stem cells making them more suitable for tissue engineering since apart from easy availability they acquire some other advantages also like the capacity to stabilize blood vessels, regulate angiogenesis and immunological response [48]. Micro-fabrication techniques (Microfluidics and micropatterning) have enabled to development of size-controlled vascularized organoids for a higher throughput at a very low cost when compared to other methods [43]. Still developing a vascularized tissue-engineered organoid for organ transplantation and other translational application remains a challenge. To overcome the limitation of low or lack of reproducibility in organoids, the use of stem cell niche was proposed as an important feature [17]. The Challenge of heterogeneity has now been resolved in iPSCs by the use of isogenic controls generated by using CRISPR-cas 9 technology [34].

### Approaches to overcome vascularization limitations

**Fig. 3:**
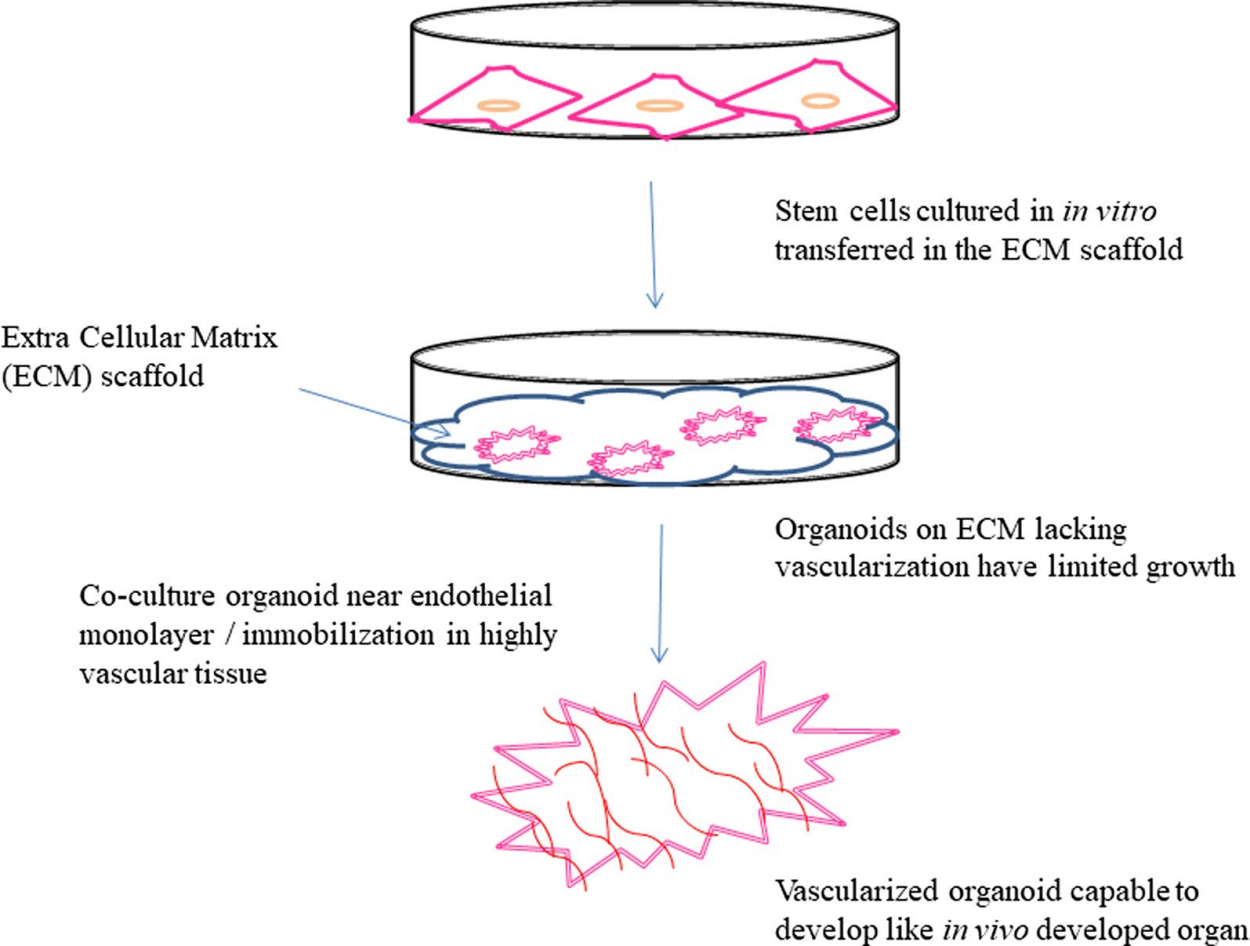
Vascularized organoid development from stem cells on the extracellular matrix (ECM) scaffold by co-culturing with endothelial cells or immobilizing the developed organoid in highly vascular tissue

The construction of organoid models is limited to a small size (a few millimeters in thickness) due to the lack of vascularization which restricts nutrient perfusion and mass transport. Therefore vascularization has been a topic of high interest for researchers for more than a decade now. The approaches adopted so far to improve vascularization have been discussed in Table 3 (Fig. 3). Bioreactor designs such as Rotating bioreactor and perfusion bioreactor have been developed to address the issues of vascularization in tissue engineering but the constructs formed using bioreactor designing fail to develop a vasculature that can support the anastomosis [49].

**Table 3:** Methods to develop vascularization, their advantages and disadvantages

Method	Process	Advantage	Disadvantage	References
*Scaffold Functionalization*				
Growth factor delivery system (VEGF, bFGF, PDGF, TGF, Angiopoietin-1 and 2)	Most basic and simple method is to load or coat the growth factor of interest to the scaffold	Pre encapsulation method ensured a prolonged release of growth factors providing high degree of vascularization	Short effective half-life due to their poor stability or fast blood clearance	[49, 50]
	Protein Modification techniques		High concentration use can induce cancer development	
	Pre-encapsulation of growth factors in dual drug delivery systems of micro or nanospheres before embedding into a scaffold			
Engineered scaffold designing	Channeled scaffold prepared by incorporating phosphate based glass fibers into collagen scaffolds or by laser cutting technique	Oxygen diffusion rate, cell alignment and angiogenesis may be controlled	**-**	[49]
	Micro patterning and molecular gradients	Improved cell viability		
*Cell Based Techniques*				
Endothelial cell co-culture	Endothelial cells introduced in the tissue via 3D multicellular spheroids or simple mixing of cultures (co-culturing)	Lumenized capillary like network develop	Functional anastomosis into host vasculature remain unsolved	[49]
Growth factor producing cells (Mesenchymal Stem Cells)	Growth factors like VEGF secreted in vivo models	Improve angiogenesis	Heterogeneity nature of MSCs and individual to individual variance major limitation that delay clinical translation of MSCs	[49, 51]
	Transfection of human MSCs with VEGF-plasmid coated scaffolds			
iPSCs	Co culture of hiPSC-endothelial cells and hiPSC-derived pericytes/MSCs led to development of tube like structure	Un-exhaustible cell source to form pre-vascularized systems	High chances of tumor formation due to the ‘unsafe’ iPSC lines and residual undifferentiated iPSCs in final product	[51]
*Pre-vascularization*				
Scaffold vessel formation	Cells (generally endothelial cells) are seeded in the scaffold to form vessel like structure before implantation	Therapeutic angiogenesis can occur in a very short period of time	Endothelial cells lack high proliferative turnover in-vitro so cannot always be cultured in therapeutic quantities	[50]
Cell sheet technology	Cells seeded on a smart cell culture substrate (example—temperature responsive substrate) to produce a 2D sheet of pre vascularized tissue	No requirement of a preexisting scaffold	-	[50, 52]
		Rapid wound healing		
In vivo bioreactor system	A scaffold implanted subcutaneously for a period of time to allow neovascularization. Flap technique and AV-loop are two important techniques for in-vivo pre-vascularization	Increased cell survival, proliferation, and vascular infiltration	Inappropriate porous microstructure	[50]

### Organoids in development of personalized medicine

**Fig. 4:**
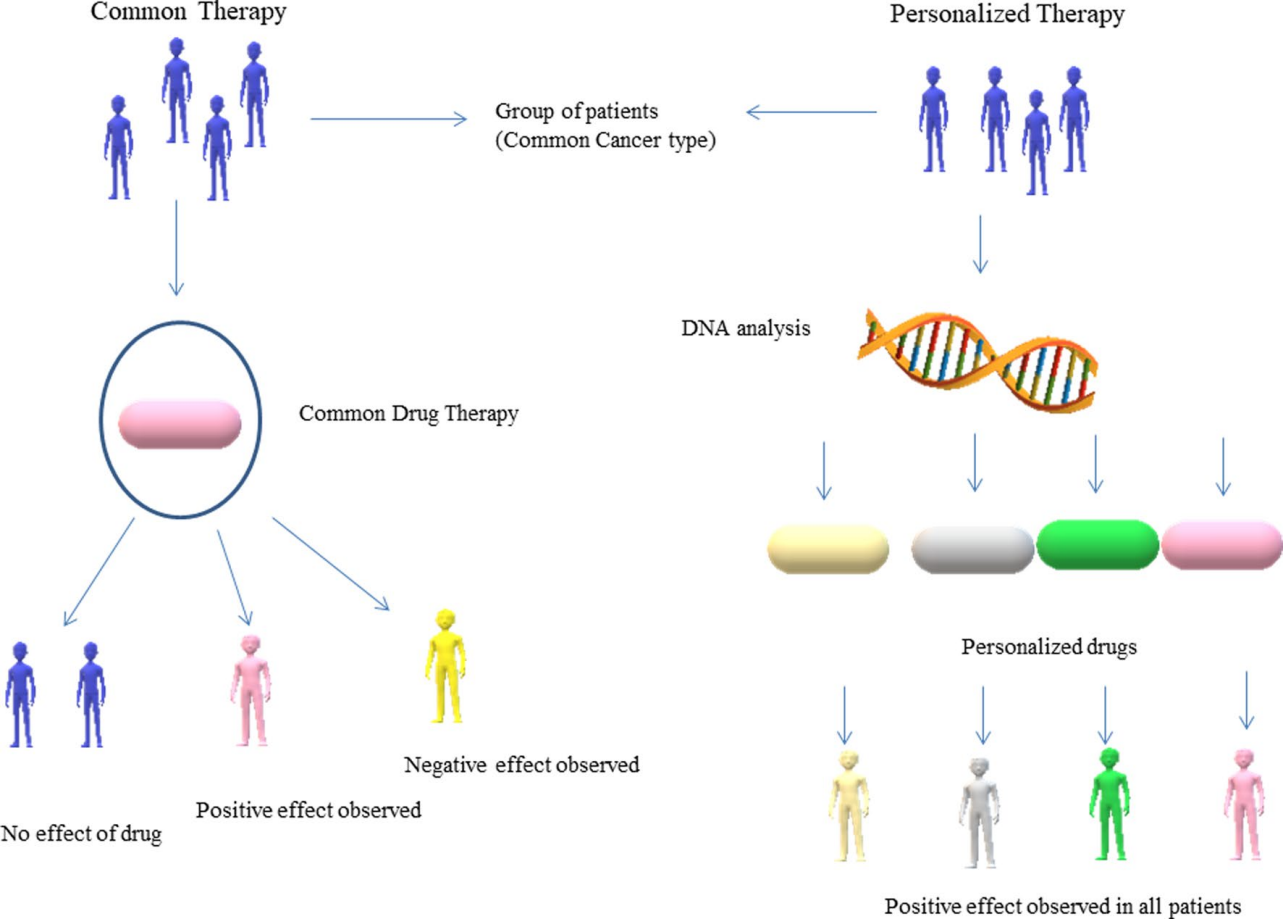
Comparison between common therapy and personalized therapy: a single drug for a particular disease when administered by a group of people would show highly variable effects. Personalized drugs customized based on genetic analysis and tested on disease models provide a clear effectivity rate of the drug

**Fig. 5:**
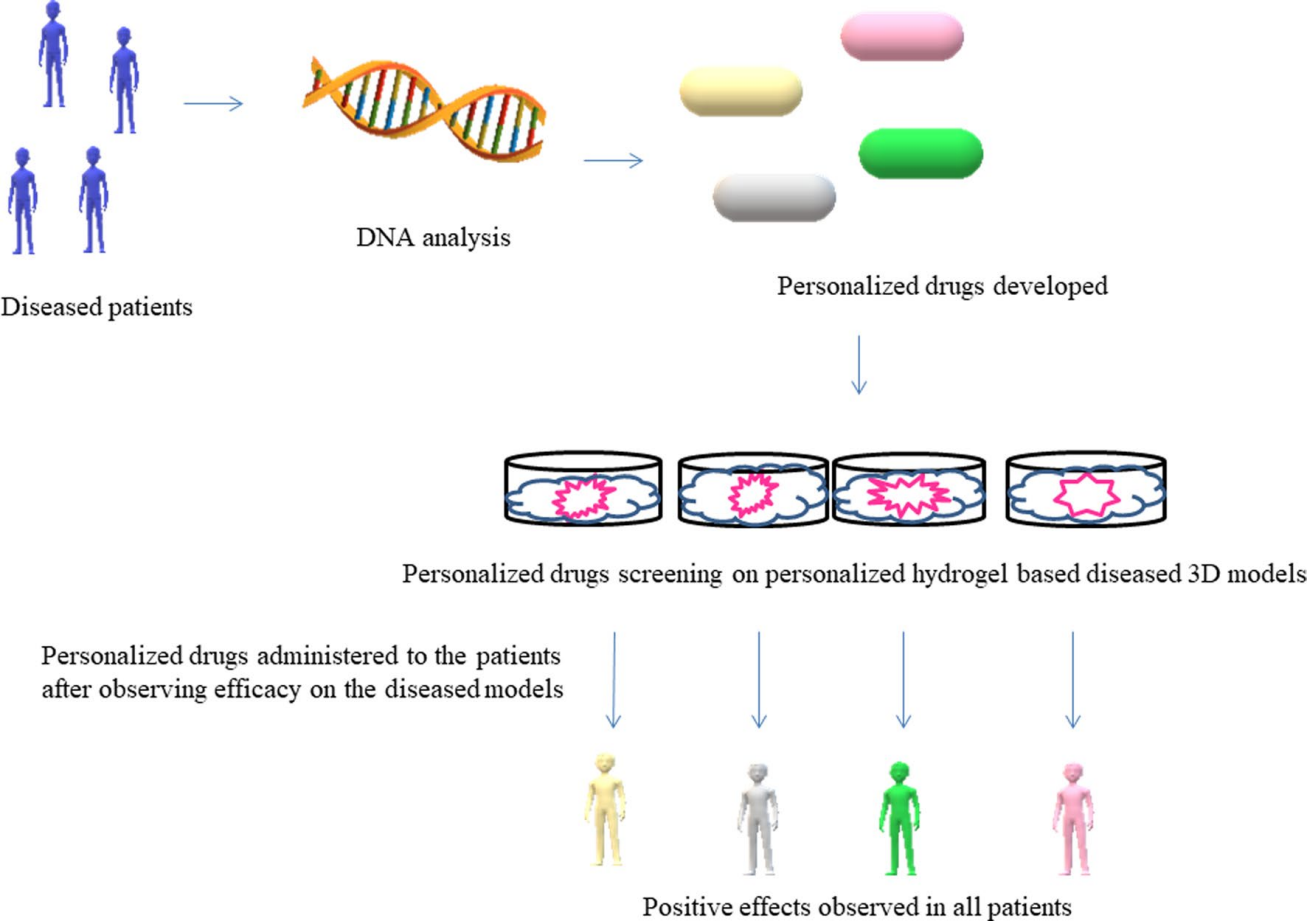
Drug screening on in vitro hydrogel-based diseased 3D micro models can provide accurate data about the efficacy of the personalized drugs designed

Personalized medicines also known as precision medicine are in great demand for the treatment and prevention of various diseases occurring due to genetic and molecular abnormalities. Cancer and neurodegenerative diseases like Alzheimer’s, Parkinson's, and Dementia are a few of the many diseases occurring due to genetic malfunctioning. Precision medicines are patient-specific (refer to Fig. 4) and developed by analyzing the patient's genetic, environmental, and lifestyle factors. Cancer treatment is challenging because the cancer cells are immortal and cancer patients have a short time for survival and since every cancer is different, it may not be possible that a single drug shows the same effect when administered by two patients suffering from the same type of cancer. Also, the cancer cells can mutate and become drug-resistant and such cells may start developing aggressively. The one-size-fits-all approach fails in cancer treatment and therefore now personalized drug therapy is being explored to find an effective cure and patient-derived organoids are a promising tool to screen an effective drug with high efficacy in a short time (refer to Fig. 5). Molecular techniques such as Next-generation sequencing (NGS), mRNA sequencing, ChIP-sequencing, and mass spectrometry (MS) are generally used to determine the genome profile of cancer cells and patients and analyze the known mutation for treatment with available anticancer drugs [44]. 3D organoid culture of the patient-derived tumor cells can help to screen early drug resistance in cells before the clinical relapse so that the second panel of drugs can be screened to early select a new therapy [45]. Magnetic 3D bioprinting approach provided routine, feasible, scalable, and fine spatial controlled 3D models developed in flat-bottom microplates, that can show high throughput applications in cancer drug discovery and personalized medicine [46]. A 3D micro-scale perfusion-based two-chamber tissue model was developed on a polymer chip where two separate chambers were containing porous polymeric scaffolds for liver and cancer cell cultures, respectively and this system was used to determine the cytotoxicity of anticancer drugs [47]. The Patient-derived organoids can be preserved in biobanks for future studies or for the use of other patients in urgent need having the same human leukocyte antigen profile so there would be no immune rejection. 3D bioprinting is a significant technology developed to control infectious diseases by providing rapid and automated production of tissue or organoid models and personalized medicine [57]. Many researchers have successfully developed brain, skin, pancreatic, breast, and other tumor constructs with microscale precision using 3D bioprinting technology for a better understanding of cancer biology and developing precise personalized anticancer drugs [58].

### Mathematical modeling and 3D cell culture

Repeatable generation of invitro engineered tissues is a challenge that can be overcome with the help of mathematical modeling, defining input parameters that yield predictable output measures of tissue maturation [63]. A mathematical model based on multiphasic mixture theory was developed to explore the interactions between molecular transport, polymer degradation, and tissue growth [64]. The swelling characteristics of hydrogels can also be measured using mathematical expressions (1) and (2) [65].

1 $${\text{Swelling}} = W_{{\text{s}}} {-}W_{{\text{d}}} /W_{{\text{d}}}$$

where *W*_s_ is the weight of hydrogel in swollen state and *W*_d_ is the weight of hydrogel in the dry state.

2 $${\text{Swelling}} = C/B\,*\,100$$

where *C* is the weight of hydrogel obtained after drying and *B* is the weight of the insoluble portion after extraction with water.

### Induced pluripotent stem cells (iPSCs) role in developing personalized medicine

Organoids are developed using embryonic stem cells or adult stem cells. The use of embryonic stem cells is limited due to ethical issues because life is sacrificed to extract these cells. The human induced pluripotent stem cells (iPSCs) development technique is now a potential alternative to the use of embryonic stem cells in organoid culture. The iPSCs are the reprogrammed somatic cells. The Yamanaka factors were over-expressed in the somatic cell derived from adult tissues which induced transformation of somatic cells into pluripotent stem cells and therefore named induced pluripotent stem cells (iPSCs). During the development of organoid using iPSCs, initially, an organoid bud develops and later the iPSCs are exploited to undergo further differentiation to form a desired mature organoid structure consisting of different cell types connected with proper cell–cell interactions [34]. An iPSC line once cultured successfully from a patient, can be preserved for a lifetime and different tissue models can be developed [34]. CRISPR-cas 9 is a genome editing technique where cas 9 is an RNA-guided nuclease. Recently CRISPR-cas 9 gene editing has been used to correct single disease-causing mutations in iPSC-based organoids. Other genome editing techniques such as zinc finger nucleases, transcription activator-like receptor nucleases (TALENs), and adeno-associated viruses can also be used for these cells [39]. In intestinal organoids derived from patients with cystic fibrosis (casual mutation in CFTR gene), first gene correction using CRISPR—cas 9 was performed, and the resulting organoids presented functional restoration as assessed in forskolin-induced swelling assays and later iPSCs derived retinal organoids from patients with retinitis pigmentosa were also gene-corrected and this approach demonstrated the potential of using gene-editing technologies for clinical transplantation [32]. In the prevention and treatment of cardiovascular disease, the myocardium is the target and design and development of Myocardium-on-chip (MOC) device using 3D cell-laden hydrogel constructs and hiPSCs derived myocardial cells enables personalized medicine studies where the individual drug response of patients with different genetic backgrounds can be tested [40]. The brain is a very important part of the human body. The blood–brain-barrier (BBB) strictly regulates the entry of solutes from the blood into the brain and this barrier is disrupted in several neurological disorders including multiple sclerosis, stroke, epilepsy, and Alzheimer’s disease therefore a neurovascular unit was developed using organ-on-chip technology and hiPSC—derived tissue that recapitulates complex BBB functions, provides a platform for modeling inheritable neurological disorders, and advance drug screening, as well as personalized medicine [41]. Blood disorder disease models for diseases such as sickle cell anemia, Fanconi anemia, Myeloproliferative neoplasm, Juvenile myelomonocytic leukemia, and Congenital megakaryocytic thrombocytopenia have been successfully developed using iPSCs and provide a positive approach towards their cure [42].

### Applications of hydrogel based organoids

Fatehullah A, et al. reviewed the potential of organoids to be used as in vitro model for studying human development and human diseases, and the near-physiological growth and self-renewing capability of organoids proved them as an excellent model system for both basic as well as translational research [29]. Decembrini et al. worked to discover a new tissue engineering approach that would standardize and accelerate the production of retinal organoids and therefore seeded mouse embryonic stem cells (mESC) on hydrogel milliwell arrays and overcoming the shortcomings of previous methods observed the rapid generation of the retina like tissue from mESC aggregates in a highly efficient and stereotypical manner [30]. Angus HCK, et al. reviewed the use of intestinal organoids for immunological research and concluded that since human colonic and small intestinal tissues are difficult to obtain and also have a short life span, intestinal organoids are the potential solution to these limitations and can act as a major tool for intestinal research [31]. The brain organoids are the most relevant alternative to the animal model for cellular and molecular processes studies of neurological diseases like Parkinson's disease, Alzheimer's disease, and autism spectrum disorders [32]. Fatal liver diseases like Cirrhosis, Biliary atresia, and Fulminant hepatic failure have liver transplantation as the only cure but due to the lack of donors, it has been a challenge so far. The polyisocyanopeptide (PIC) has proven to be a potential hydrogel for developing and maintaining human liver organoids and also it can be used for various other clinical applications [33]. Human respiratory tract organoids are suitable models to study various respiratory viruses like respiratory syncytical virus (RSV), influenza viruses, and coronavirus (SARS-CoV-2) [34]. A breakthrough in the pathogenesis study of Zika virus infection was achieved only after brain organoids were successfully cultured and included for research perspective and the organoid model of zika virus infection also helped to develop a potential treatment [38]. The past research on hydrogel and hydrogel-based organoids have enabled researchers today to use hydrogel-based organoids in therapeutics and drug development, tissue engineering, cancer research, regenerative medicine, stem cell study, and molecular mechanism of various human diseases

## Conclusions

The continuous research for decades in the field of animal cell culture has led to many advancements and 3D cell culture has proven as a huge success in the process so far. 3D cultures resemble well to the original organ therefore nowadays they have a high preference in various clinical applications.

Hydrogels play an important role in developing a biocompatible and bioactive 3D culture system therefore natural hydrogels should be preferred, plant-based natural hydrogels being the most preferable because animal-based natural hydrogels have a risk of contamination and batch to batch variability. Hydrogel-based organoid culture has presented a new direction to the researchers to understand and unveil the human body mechanisms, remaining unknown. An organoid is a miniaturized model of an organ and bridges the gap between conventional 2D cell culture and complex animal models. In the last few years, 3D bioprinting has received high acceptance in cell culture research and has proven a valuable technology to develop vascularized 3D models that can be used for rapid drug screening, personalized medicine, toxicity evaluation, tissue engineering, and regenerative medicine.

Despite achieving a level of physiological resemblance there have been a few limitations with organoids like limited proliferation and differentiation rate, small size, and insufficient nutrient accessibility that have restricted their application to drug discovery and regenerative medicine. Overcoming these limitations in the future can help to completely supplant the requirement of future animal models for various clinical researches. Also, the organoid cultures have the potential to compensate for the exigency of donors in organ transplantation and there would be negligible immune rejection possibilities.

## Future scope

Vascularization is been studied for a long time now but the approaches developed so far does not guarantee successful results on transitioning from in-vitro to in-vivo conditions in human being. To overcome the limitation of vascularization, the transdifferentiation phenomenon can be explored by researchers. It is the process of conversion of one type of cell into a completely another type of cell by introducing some transcriptional factors or extracellular growth factors or sometimes both can be worked together. One approach can be to induce transdifferentiation in a developing organoid while monitoring the nutrient supply and oxygen diffusion rate to develop a high-quality vascular system.

## Data Availability

Not applicable.

## Abbreviations

2D: Two dimensional
3D: Three dimensional
SCPL: Solvent casting and Particle Leaching
HRP: Horseradish peroxidase
H_2_O_2_: Hydrogen peroxide
BMSCs: Bone-marrow-derived mesenchymal stem cells
pl: Picolitre
CaCl_2_: Calcium Chloride
LAB: Laser-assisted bioprinting
iPSCs: Induced Pluripotent Stem Cells
TALEN: Transcription activator-like receptor nucleases
CFTR: Cystic fibrosis transmembrane conductance regulator
MOC: Myocardium-On-Chip
hiPSCs: Human-induced Pluripotent Stem Cells
BBB: Blood–Brain-Barrier
mESC: Mouse Embryonic Stem Cell
PIC: Polyisocyanopeptide
RSV: Respiratory Syncytical Virus
SARS-CoV-2: Severe acute respiratory syndrome coronavirus 2
VEGF: Vascular endothelial growth factor
bFGF: Basic fibroblast growth factor
PDGF: Platelet-derived growth factor
TGF: Transforming growth factor
MSCs: Mesenchymal stem cells

## References

[1] Antoni D, Burckel H, Josset E, Noel G (2015). Three-dimensional cell culture: a breakthrough in vivo. Int J Mol Sci.

[2] Rodriguez-Hernandez CO (2014). Cell culture: history, development and prospects. Int J Curr Res Aca Rev.

[3] Jensen C, Teng Y (2020). Is it time to start transitioning from 2D to 3D cell culture?. Front Mol Biosci.

[4] Permlid AM, Roci P (2019). Unique animal friendly 3D culturing of human cancer and normal cells. Toxicol In Vitro.

[5] Eltom A, Zhong G, Muhammad A (2019). Scaffold techniques and designs in tissue engineering functions and purposes: a review. Adv Mater Sci Eng.

[6] Akther F, Little P, Li Z (2020). Hydrogels as artificial matrices for cell seeding in microfluidic devices. RSC Adv.

[7] Ahmed EM (2015). Hydrogel: preparation, characterization, and applications: a review. J Adv Res.

[8] Worthington P, Pochan DJ, Langhans SA (2015). Peptide hydrogels—versatile matrices for 3D cell culture in cancer medicine. Front Oncol.

[9] Caliari S, Burdick JA (2016). A practical guide to hydrogels for cell culture. Nat Methods.

[10] Swetha S, Lavanya K, Sruthi R, Selvamurugan N (2020). An insight into cell-laden 3D-printed constructs for bone tissue engineering. J Mater Chem B.

[11] Goubko CA, Basak A, Majumdar S, Cao X (2014). Dynamic cell patterning of photoresponsive hyaluronic acid hydrogels. J Biomed Master Res Part A.

[12] Xu K, Fu Y, Chung W, Zheng X (2012). Thiol-ene-based biological/synthetic hybrid biomatrix for 3-D living cell culture. J Acta Biomater.

[13] Bedard P, Gauvin S, Ferland K (2020). Innovative human three-dimensional tissue-engineered models as an alternative to animal testing. Bioengineering.

[14] Zanoni M, Cortesi M, Zamagni A (2020). Modeling neoplastic disease with spheroids and organoids. J Hematol Oncol.

[15] Brown AC, Barker TH (2014). Fibrin-based biomaterials: modulation of macroscopic properties through rational design at the molecular level. Acta Biomater.

[16] Langhans SA (2018). Three-dimensional in vitro cell culture models in drug discovery and drug repositioning. Front Pharmacol.

[17] Chenchula S, Kumar S, Babu S (2019). Comparitive efficacy of 3dimensional (3D) cell culture organoids vs 2dimensional (2D) cell cultures vs experimental animal models in disease modeling, drug development, and drug toxicity testing. Int J Cur Res Rev.

[18] Ravi M, Paramesh V, Kaviya SR (2014). 3D cell culture systems—advantages and applications. J Cell Physiol.

[19] Tibbitt MW, Anseth KS (2009). Hydrogels as extracellular matrix mimics for 3D cell culture. Biotechnol Bioeng.

[20] Mohammadinejad R, Maleki H (2019). Status and future scope of plant-based green hydrogels in biomedical engineering. Appl Mater Today.

[21] Pan Z, Ye H, Wu D (2021). Recent advances on polymeric hydrogels as wound dressings. APL Bioeng.

[22] Mantha S, Pillai S, Khayambashi P (2019). Smart hydrogels in tissue engineering and regenerative medicine. Materials.

[23] Nezhad-Mokhtari P, Ghorbani M (2019). Chemical gelling of hydrogels-based biological macromolecules for tissue engineering: photo- and enzymatic-crosslinking methods. Int J Biol Macromol.

[24] Zhu C, Lei H, Fan D, Duan Z (2018). Novel enzymatic crosslinked hydrogels that mimic extracellular matrix for skin wound healing. J Mater Sci.

[25] Zhang Y, Chen H, Zhang T (2018). Injectable hydrogels from enzyme-catalyzed crosslinking as BMSCs-laden scaffold for bone repair and regeneration. Msc.

[26] Zhang YS, Khademhosseini A (2017). Advances in engineering hydrogels. Science.

[27] Seol YJ, Kang HW (2014). Bioprinting technology and its applications. Eur J Cardiothorac Surg.

[28] Murphy SV, Atala A (2014). 3D bioprinting of tissues and organs. Nat Biotechnol.

[29] Fatehullah A, Tan SH, Barker N (2016). Organoids as an in vitro model of human development and disease. Nat Cell Biol.

[30] Decembrini S, Hoehnel S (2020). Hydrogel-based milliwell arrays for standardized and scalable retinal organoid cultures. Sci Rep.

[31] Angus HCK, Butt AG, Schultz M, Kemp RA (2020). Intestinal organoids as a tool for inflammatory bowel disease research. Front Med.

[32] Hofer M, Lutolf MP (2021). Engineering organoids. Nat Rev Mater.

[33] Ye S (2020). A chemically defined hydrogel for human liver organoid culture. Adv Funct Mater.

[34] Kim J, Koo BK, Knoblich JA (2020). Human organoids: model systems for human biology and medicine. Nat Rev Mol Cell Biol.

[35] Hunter P (2014). One organ at a time. EMBO Rep.

[36] Hollander A, Macchiarini P, Gordijn B, Birchall M (2009). The first stem cell-based-tissue-engineered organ replacement: implications for regenerative medicine and society. Regen Med.

[37] Grebenyuk S, Ranga A (2019). Engineering organoid vascularization. Front Bioeng Biotechnol.

[38] Li Y, Tang P, Cai S, Peng J, Hua G (2020). Organoid based personalized medicine: from bench to bedside. Cell Regen.

[39] Mou H, Brazauskas K, Rajagopal J (2015). Personalized medicine for cystic fibrosis: establishing human model systems. Pediatr Pulmonol.

[40] Ellis BW, Acun A, Can UI, Zorlutuna P (2015). Human iPSC-derived myocardium-on-chip with capillary-like flow for personalized medicine. Biomicrofluidics.

[41] Vatine GD, Barrile R, Workman MJ (2019). Human iPSC-derived blood-brain barrier chips enable disease modeling and personalized medicine. Cell Stem Cell.

[42] Kim C (2014). Disease modeling and cell based therapy with iPSC: future therapeutic option with fast and safe application. Blood Res.

[43] Velasco V, Shariati SA, Esfandyarpour R (2020). Microtechnology-based method for organoid models. Microsyst Nanoeng.

[44] Popova AA, Levkin PA (2020). Precision medicine in oncology: in vitro drug sensitivity and resistance test (DSRT) for selection of personalized anticancer therapy. Adv Ther.

[45] Silvestri A, Schumacher D, Silvestrov M (2017). In vitro three-dimensional cell cultures as tools for precision medicine. Mech Mol Carcinog.

[46] Souza G (2020). Magnetic 3D bioprinting for personalized medicine. Cytotherapy.

[47] Ma L, Barker J, Zhou C, Li W (2012). Towards personalized medicine with a three-dimensional micro-scale perfusion-based two-chamber tissue model system. Biomaterials.

[48] Avolio E, Alvino VV, Ghorbel MT, Campagnolo P (2017). Perivascular cells and tissue engineering: current applications and untapped potential. Pharmacol Ther.

[49] Lovett M, Lee K, Edwards A, Kaplan DL (2009). Vascularization strategies for tissue engineering. Tissue Eng Part B Rev.

[50] Amirsadeghi A, Jafari A, Eggermont LJ (2020). Vascularization strategies for skin tissue engineering. Biomater Sci.

[51] Yang G, Mahadik B, Choi JY, Fisher JP (2020). Vascularization in tissue engineering: fundamentals and state-of-art. Prog Biomed Eng.

[52] Kiaie N, Gorabi AM, Ahmadi Tafti SH, Rabbani S (2020). Pre-vascularization approaches for heart tissue engineering. Regen Eng Transl Med.

[53] Dey M, Ozbolat IT (2020). 3D bioprinting of cells, tissues, and organs. Sci Rep.

[54] Farhat J, Pandey I, AlWahsh M (2021). Transcending toward advanced 3D-cell culture modalities: a review about an emerging paradigm in translational oncology. Cells.

[55] Benwood C, Chrenek J, Kirsch RL, Masri NZ (2021). Natural biomaterials and their use as bioinks for printing tissues. Bioengineering.

[56] Gillispie G, Prim P, Copus J, Fisher J (2020). Assessment methodologies for extrusion-based bioink printability. Biofabrication.

[57] Yi HG, Kim H, Kwon J (2021). Application of 3D bioprinting in the prevention and the therapy for human diseases. Sig Transduct Target Ther.

[58] Augustine R, Kalva SN, Ahmad R (2021). 3D Bioprinted cancer models: revolutionizing personalized cancer therapy. Transl Oncol.

[59] Curvello R, Kerr G (2021). Engineered plant-based nanocellulose hydrogel for smallintestinal organoid growth. Adv Sci.

[60] Lopez-Marcial GR, Zeng AY, Osuna C (2018). Agarose-based hydrogels as suitable bioprinting materials for tissue engineering. ACS Biomater Sci Eng.

[61] Indurkar A, Pandit A, Jain R, Dandekar P (2021). Plant-based biomaterials in tissue engineering. Bioprinting.

[62] Mahendiran B, Muthusamy S, Sampath S (2021). Recent trends in natural polysaccharide based bioinks for multiscale 3D printing in tissue regeneration: a review. Int J Biol Macromol.

[63] Price JC, Krause AL (2020). Predicting bone formation in mesenchymal stromal cell—seeded hydrogels using experiment-based mathematical modeling. Tissue Eng Part A.

[64] Akalp U, Bryant SJ, Vernerey FJ (2016). Tuning tissue growth with scaffold degradation in enzyme-sensitive hydrogels: a mathematical model. Soft Matter.

[65] Malpure PS, Patil SS (2018). A review on-hydrogel. Am J PharmTech Res.

[66] Chamkouri H, Chamkouri M (2021). A review of hydrogels, their properties and application in medicine. Am J Biomed Sci Res.

[67] Li X, Sun Q (2018). Functional hydrogels with tunable structures and properties for tissue engineering applications. Front Chem.

